# Benefits and challenges of adding BKM120 to a BI-3406 plus trametinib combination therapy

**DOI:** 10.1186/s12885-026-16409-0

**Published:** 2026-07-03

**Authors:** Benjamin Schulz, Emily Leitner, Mehrdad Rabierad, Muhammad Imran Khan, Luise Ehlers, Hugo Murua Escobar, Robert Jaster, Brigitte Vollmar, Dietmar Zechner

**Affiliations:** 1 Rudolf-Zenker-Institute of Experimental Surgery, University Medical Centre Rostock, Rostock, Germany; 2 Department of Internal Medicine, Division of Gastroenterology, Hepatology and Nutritional Medicine, University Medical Centre Rostock, Rostock, Germany; 3 Institute of Medical Genetics, University Medical Centre Rostock, Rostock, Germany; 4 Comprehensive Cancer Center – Mecklenburg Vorpommern, Universitätsmedizin Rostock, Rostock, Germany

**Keywords:** Therapeutic limitations, Distress of chemotherapy, Buparlisib, Immune checkpoint, Targeted therapies, Pancreatic ductal adenocarcinoma, KRAS G12D mutation

## Abstract

**Background:**

Achieving a balance between efficacy and side effects is essential for the success of new combinatorial cancer therapies. This study investigated the impact of adding BKM120, a PI3K inhibitor, to a dual regimen consisting of BI-3406, a KRAS/SOS1 inhibitor, and trametinib, a MEK inhibitor.

**Methods:**

The therapeutic effects of these drug combinations were evaluated in the pancreatic cancer cell line 6606PDA using both monolayer and three-dimensional cultures. Additionally, their efficacy and potential side effects were assessed in vivo using a syngeneic orthotopic mouse model of pancreatic cancer.

**Results:**

*In vitro* studies demonstrated that the addition of BKM120 to BI-3406 and trametinib significantly reduced cell viability in both monolayer and three-dimensional cultures. However, in a syngeneic pancreatic cancer mouse model, the triple therapy failed to significantly improve survival outcome compared to the dual therapy. Tumor weights were unaffected in female mice, while a minor, non-significant reduction was observed in male mice. This was accompanied by a slight, non-significant decrease in cancer cell proliferation. In the triple therapy group, *Cdkn2a* expression in tumors, a marker for senescence, remained largely unchanged. However, PD-L1 was significantly reduced in males, and CD8^+^ T-cell infiltration was notably enhanced in females. Importantly, the triple therapy demonstrated several concerning drawbacks.

It significantly increased lung metastases in female mice, reduced lymphocyte and erythrocyte counts, and elevated C-peptide concentrations in both sexes.

Furthermore, behavioral analysis indicated a significant decline in burrowing and nesting activities among female mice during specific experimental phases.

**Conclusion:**

The addition of BKM120 to the combination of BI-3406 and trametinib provides minimal therapeutic benefit while introducing significant adverse effects, underscoring the need for caution when considering clinical applications.

**Graphical Abstract:**

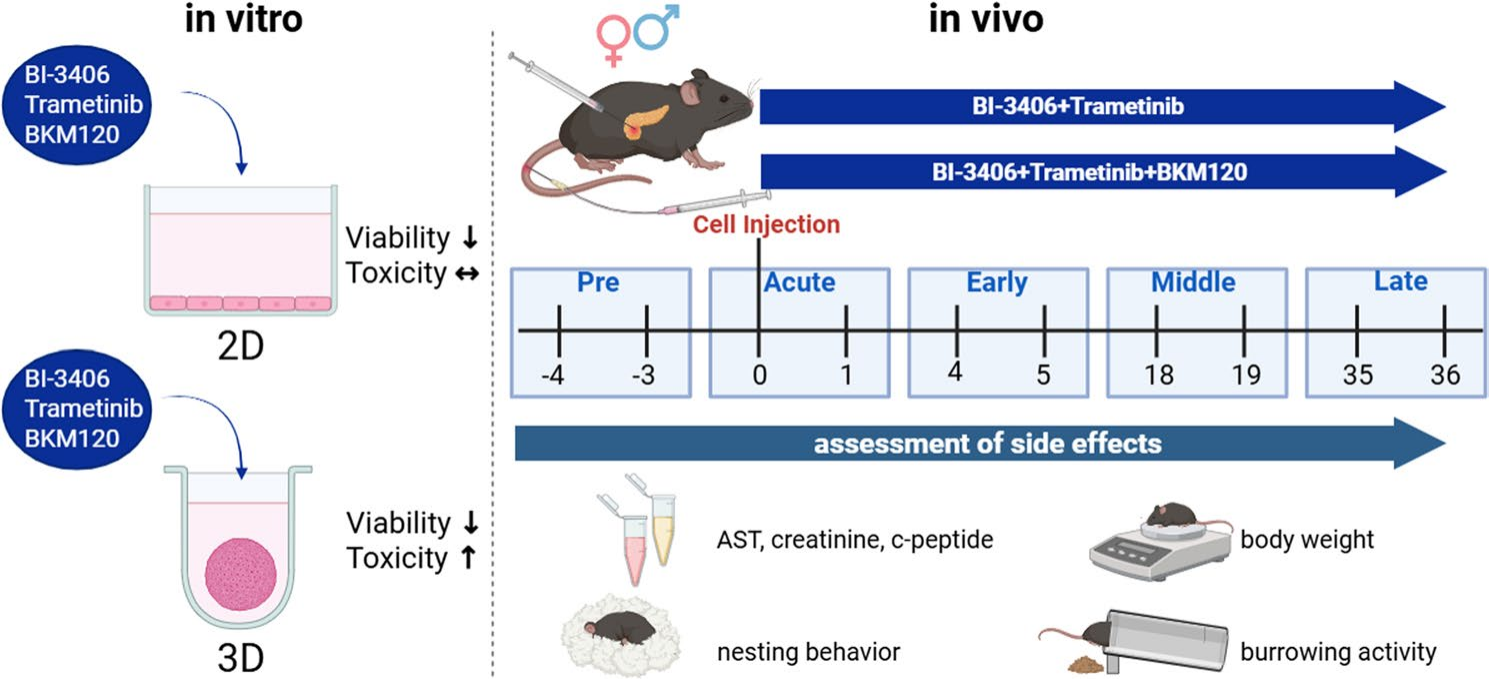

**Supplementary Information:**

The online version contains supplementary material available at 10.1186/s12885-026-16409-0.

## Background

Activating mutations in the RAS superfamily of small guanosine triphosphatase proteins, which include KRAS, HRAS, and NRAS, drive cancer cell proliferation, survival, and metabolic activity through activation of the RAF/MEK/MAPK and PI3K/Akt signaling pathways [1, 2]. These mutations are prevalent in various cancers.

For example, *KRAS* mutations occur in approximately 90% of ancreatic ductal adenocarcinomas (PDAC), 45% of olorectal cancer, and 35% of ung adenocarcinomas in the United States [3]. Historically, RAS proteins were deemed “undruggable” for over four decades. However, recent advancements have renewed efforts to therapeutically target *RAS*, leading to the development of promising drugs [3].

Recently, the FDA has approved the first drugs targeting specific RAS mutations.

Sotorasib, which inhibits the *KRAS G12C* mutation, was approved in 2021 for patients with advanced non-small cell lung cancer [4]. Adagrasib, another KRAS G12C inhibitor, received approval in 2022 [5]. However, *KRAS G12C* mutations are present only in a subset of cancers. For instance, just 1.5% of DAC patients harbor this mutation. Conversely, KRAS G12D is the most frequent KRAS mutation in PDAC (39% of ases), followed by G12V and G12R mutations [6]. To address a broader spectrum of RAS-driven cancers, additional drugs targeting multiple *KRAS* mutations are in development. One such approach is to inhibit SOS1, a guanine exchange factor important for KRAS activation. SOS1 inhibitors, such as MRTX0902 and BI-3406, disrupt the KRAS-SOS1-interaction, thereby reducing RAS signaling and limiting tumor cell proliferation [7, 8].

Despite the success of early RAS-targeting drugs, achieving a durable therapeutic response remains a challenge, as cancer cells often adapt by reactivating pathways such as the MAPK pathway after RAS inhibition [9–11]. To overcome these adaptive mechanisms, recent studies have investigated strategies for vertical inhibition of multiple components of the RAS/RAF/MEK/MAPK signaling pathway by using combination therapies [8, 12–14]. For example, combining SOS1 inhibition (BI-3406) with the MEK inhibitor trametinib demonstrated strong synergy and tumor regression in several KRAS-mutant cancer models [8]. In addition to vertical pathway targeting, horizontal inhibition of parallel signaling pathways has also shown promise [13]. For instance, the combined inhibition of the RAF/MEK/MAPK and PI3K/AKT pathways synergistically suppressed melanoma cell lines and improved progression-free survival in patients with metastatic melanoma [15, 16]. Building on this approach, we previously demonstrated that the triple therapy combining the SOS1 inhibitor BI-3406, the MEK inhibitor trametinib, and the pan-PI3K inhibitor BKM120 (buparlisib) exhibited significant anticancer effects in various PDAC cell lines [17]. In a first *in vivo* proof of concept study, we could also demonstrate that this triple therapy can reduce tumor size in a PDAC mouse model [18].

In this study, we carefully evaluate the benefits and challenges of adding BKM120 to the BI-3406 plus trametinib combination therapy.

## Methods

### Cell culture and *in vitro* analysis

The murine 6606PDA cell line, harbouring the KRas G12D mutation was kindly provided by Prof. Tuveson (Cold Spring Harbor Laboratory, USA) [19]. Cells were routinely cultured in DMEM (4.5 g/L Glucose, PAN Biotech GmbH, Aidenbach, Germany) supplemented with 10% ftal calf serum (PAN Biotech GmbH) and penicillin/streptomycin (100 U/ml, PAN Biotech GmbH). To assess the effects of BI-3406 (provided by Boehringer Ingelheim via their Open Innovation Portal opnMe), trametinib, and BKM120 (both purchased from Chemietek, Indianapolis, USA) on cell viability and cytotoxicity, either alone or in combination, cells were seeded at a density of 5 × 10³ in 96-well plates (Greiner Bio-One GmbH, Frickenhausen, Germany). The substances were dissolved in DMSO and added at the specified concentrations, followed by a 48-hour incubation. Cytotoxicity and cell viability were measured using the CellTox™ Green cytotoxicity assay and CellTiter-Glo^®^ luminescent cell viability assay (Promega GmbH, Walldorf, Germany). For 3D culture, 6606PDA cells were seeded at a density of 3 × 10⁴ in 96-well cell-repellent plates (650970, Greiner Bio-One GmbH) and cultured for four days until spheroid formation.

The spheroids were then treated, and viability and cytotoxicity were assessed as described earlier. For Western blot analysis, 6606PDA cells were seeded at a density of 4 × 10⁵ or 2 × 10⁵ cells per well in a six-well plate. The following day, cells were treated with either vehicle or 1 µM BKM120 for 6–24 h. Protein extracts (30 µg) were analysed using a rabbit anti-p70 S6 kinase (phospho-T389) antibody (dilution: 500×, ab2571, Abcam, Cambridge, United Kingdom) and a mouse anti-β-actin antibody (dilution: 20000×, A5441, Sigma-Aldrich, St. Louis, Missouri, USA) as primary antibodies.

### Animals

C57BL/6J mice used in this study were bred under specified pathogen-free conditions in our animal facility. The health status of the animal stock was routinely monitored, and any mice testing positive for *Helicobacter sp.*,* Rodentibacter heylii*, or murine norovirus were excluded from the study. Throughout the experiment, all mice were kept in single-housed type III cages (Zoonlab GmbH, Castrop-Rauxel, Germany) with a 12-hour light-dark cycle, at a temperature of 21 ± 2 °C, and relative humidity of 60 ± 20%. Food (pellets, 10 mm, ssniff-Spezialdiäten GmbH, Soest, Germany) and tap water were provided *ad libitum*. Environmental enrichment included nesting material (shredded tissue paper, Verbandmittel GmbH, Frankenberg, Germany), a paper roll (75 × 38 mm, H 0528–151, ssniff-Spezialdiäten GmbH), and a wooden stick (40 × 16 × 10 mm, Abedd, Vienna, Austria).

### Pancreatic cancer model and therapeutic intervention

Male and female C57BL/6J mice, aged 16 to 28 weeks, were anesthetized with 1–3 vol% isoflurane, and perioperative analgesia was provided via subcutaneous injection of carprofen (5 mg/kg). Eye ointment was applied to prevent dryness, and during surgery, the mice were kept warm on a heating plate at 37 °C. Each mouse received both an orthotopic and an intravenous cell injection. For the intravenous injection, 6606PDA cells were resuspended in Hank’s Balanced Salt Solution (PAN Biotech GmbH, Aidenbach, Germany), and 50 µL of the cell suspension (7 × 10⁶ cells/mL) were injected into the tail vein using a catheter (Fine Bore Polyethylene Tubing, 0.28 mm ID, 0.61 mm OD, Smiths Medical International Ltd., Hythe, UK). The orthotopic injection was performed as previously described [18]. Briefly, 2.5 × 10⁵ 6606PDA cells were suspended in 5 µL PBS/Matrigel (BD Basement Membrane Matrix, 354248, Corning Inc., New York, USA) and injected into the head of the pancreas with a 25 µL syringe (Hamilton, Reno, USA). The abdomen was then closed with two sutures (Johnson & Johnson Medical GmbH, Norderstedt, Germany), and the mice were placed in front of a heating lamp for 20–30 min. For continuous analgesia, 3000 mg/L metamizol (Novaminsulfon-ratiopharm 500 mg/mL, Ratiopharm GmbH, Ulm, Germany) was added to the drinking water daily until the end of the experiment. On day 4 after surgery, the animals were randomized into treatment groups: either a combination of BI-3406 and trametinib, or BI-3406, trametinib, and BKM120. The compounds were dissolved in a mixture of 60% Posal50PG (Lipoid GmbH, Ludwigshafen, Germany), 30% PG400 (Merck KGaA, Darmstadt, Germany), and 10% ehanol (99.6%, ndenatured). The test substances and vehicle were administered by oral gavage from day 4 until euthanasia on day 36, following a 5 days on/2 days off dosing schedule. The drug concentrations administered via gavage were: BI-3406 at 50 mg/kg (twice daily), trametinib at 0.1 mg/kg (twice daily), and BKM120 at 30 mg/kg (once daily). On day 36, following tumor cell injection, mice were gavaged with the therapy and injected with BrdU (2.5 µL/g body weight at a concentration of 20 mg/mL) 1.5–2 h before euthanasia by cervical dislocation under deep anesthesia (4–5 vol% isoflurane). Tumors and lungs were harvested and preserved in either 4% PS-buffered paraformaldehyde (PFA, Formafix GmbH, Düsseldorf, Germany), TissueTek™ (Sakura Finetek Germany GmbH, Umkirch, Germany), or snap-frozen in liquid nitrogen for later analyses. A total of 64 mice were used in the experiments, with 9 excluded due to perioperative complications.

### Assessment of animal wellbeing

Animal wellbeing was evaluated by monitoring body weight, burrowing activity, nesting behavior, and a distress score at specific time points. For instance, the distress score was evaluated on day 0 (30 min after finishing surgery), burrowing and nesting activity was observed from the evening of day 0 to the morning of day 1, and body weight was recorded on day 1 after surgery. To gain an overview of animal wellbeing throughout the experiment, these four parameters were measured before cell injection (pre phase: days − 4 to -3), during the acute (days 0 to 1), early (days 4 to 5), middle (days 18 to 19), and late (days 35 to 36) phases of the experiment.

Burrowing activity was analyzed using a 3D-printed tube (15 cm length, 6.5 cm diameter) filled with 200 g of food pellets. The tube was placed in the mouse cage 2–3 h before the dark phase, and the remaining pellets were weighed after 17 h. Nest-building behavior was evaluated by placing a cotton nestlet (5 cm square of pressed cotton batting, Zoonlab GmbH, Castrop-Rauxel, Germany) into the cage 30 to 60 min before the dark phase. Nests were scored at 9:30 a.m. ± 2 h the next day using a modified Deacon scoring system [20], with an additional 6th point defining a perfect nest: a crater-like structure with more than 90% o the nest wall circumference higher than the body height of the coiled-up mouse.

### Histology

Following fixation in 4% PBS-buffered PFA for at least 24 h, the left lung lobe was serially sectioned into 4 μm slices and stained with hematoxylin and eosin to assess metastasis. Metastasis was analyzed semi-automatically using QuPath 0.4.3.

After stain vectors were adjusted to optimize the recognition of stained tissue, the lung tissue was identified by pixel classification. After confirming accurate tissue detection, annotations were created to define the total tissue area. The metastatic lesions were delineated using the wand tool and all metastatic areas, as well as the total analyzed lung tissue were exported as annotation measurements, and the percentage of mice with metastasis was then calculated in Excel. Tumors were similarly fixed, and immunohistochemistry was performed for CD8α (100×, 4SM15-Biotin, eBioscience, San Diego, USA) with secondary detection by Streptavidin-AP (100×, Invitrogen, Waltham, USA), BrdU (50×, BU20a, Dako, Hamburg, Germany) with secondary detection by an HRP-conjugated antibody (polyclonal goat anti-mouse, 100×, Dako, Hamburg, Germany), PD-L1 (200×, D5V3B, Cell Signaling Technology, Danvers, USA) with secondary detection by an AP-conjugated antibody (goat anti-rabbit, 200×, 97048, Abcam) and T389 phosphorylated p70 S6 kinase (dilution: 50×, ab2571, Abcam) with secondary detection by an AP-conjugated antibody (goat anti-rabbit, 200×, 97048, Abcam). The detection of BrdU- and CD8-positive cells, as well as PD-L1- and phosphorylated p70 S6 kinase- positive tissue, was performed using QuPath 0.4.3.

### Quantitative real-time polymerase chain reaction (TaqMan RT-qPCR)

Tumor samples were snap-frozen in liquid nitrogen during tissue harvest. Total RNA was extracted using QIAzol lysis reagent and the RNeasy Mini Kit (both from Qiagen, Hilden, Germany). cDNA was synthesized from 100 ng of extracted RNA using the High-Capacity cDNA Reverse Transcription Kit (Applied Biosystems, Waltham, USA). RNA extracted from the lungs of four healthy female wild-type C57BL/6J mice served as the calibrator. TaqMan qPCR was conducted on a CFX Duet real-time qPCR system (Bio-Rad Laboratories GmbH, Feldkirchen, Germany) using probes (Applied Biosystems, Waltham, USA) specific for *Gapdh* (Mm99999915_g1) and *Cdkn2a* (Mm00494449_m1). Ct values were calculated using CFX Maestro software (Version 2.3, Applied Biosystems), and the data were processed using the ∆Ct method (Ct^Cdkn2a^ – Ct^Gapdh^) and normalized as ∆∆Ct (∆Ct^Calibrator^ - ∆Ct). Statistical analysis was performed using ∆∆Ct values.

### Blood chemistry

Blood samples were collected via retroorbital bleeding immediately before euthanasia, centrifuged, and the resulting plasma was stored at -80 °C for subsequent analysis. Blood chemistry parameters, such as aspartate aminotransferase (AST), and creatinine were measured using a cobas c111 system (Roche Diagnostics, Mannheim, Germany), and C-peptide levels were quantified with a mouse C-peptide ELISA kit (ALPCO, Salem, USA) according to the manufacturer’s instructions. Five healthy 19–20 weeks old C57BL/6J mice of either sex have been used as controls.

### Data presentation, statistical analysis, and use of large language models

Data are presented in Supplementary file S1 and were analyzed using GraphPad Prism (version 8.0.1, GraphPad Software Inc., San Diego, USA). Results are shown as box plots (with individual data points depicted and whiskers indicating the minimum and maximum). Please note that data based on the BI-3406, trametinib, and BKM120 triple therapy was previously published [18]. Statistical significance was determined using various methods, as detailed in the figure legends, depending on the number of independent variables and data characteristics. When analyzing the impact of two independent variables (e.g., time and therapy) on a dependent variable, a two-way repeated measures ANOVA with Sidak’s post-hoc test was employed. For a single independent variable, normality was assessed using the Shapiro–Wilk test. Comparisons between two unpaired groups were performed using the unpaired t-test (with Welch’s correction for unequal sample sizes) or the Mann–Whitney rank sum test. For comparisons involving more than two groups, either a one-way ANOVA with Dunnett’s post-hoc test or a Kruskal-Wallis test with Dunn’s post-hoc test was used. Survival analysis was conducted using the Kaplan-Meier estimator, followed by a log-rank test. Differences were considered significant at *p* < 0.05. Large language models were used for searching publications (e.g., Perplexity, SciSpace) and for improving the clarity, grammar, and syntax of some sentences in this manuscript (ChatGPT 3.5).

## Results

**Fig. 1:**
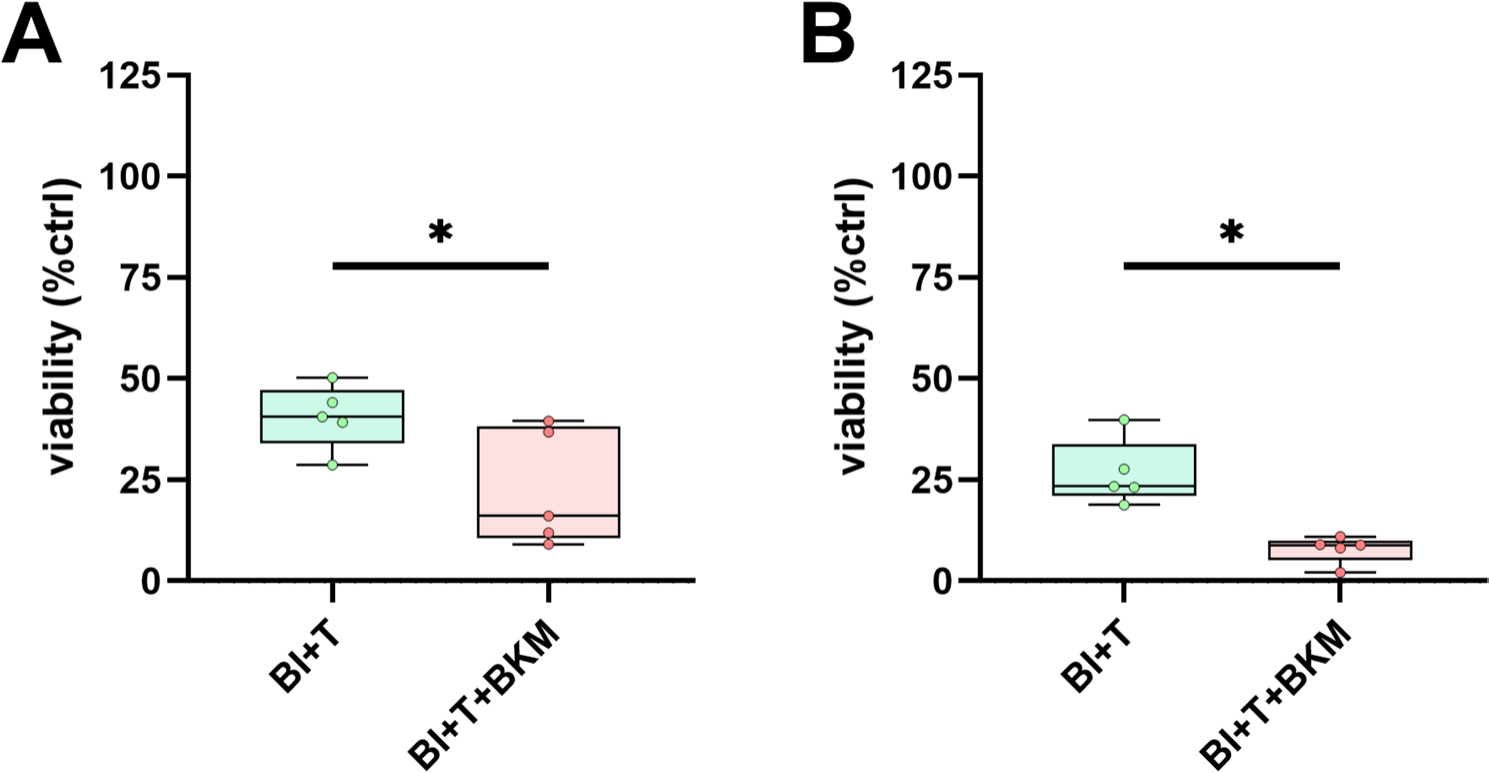
Impact of BKM120 on cell viability in combination therapy. 6606PDA cells were treated with BI-3406 (BI, 10 µM) and trametinib (T, 0.064 µM) with or without BKM120 (BKM, 1 µM) and analyzed for cell viability in two-dimensional (**A**) and three-dimensional (**B**) culture systems. Statistical analyses were performed using an unpaired t-test. *p < 0.05

In an initial proof-of-principle *in vitro* experiment, we evaluated whether adding 1µM BKM120 to a BI-3406 (10µM) and trametinib (0,064µM) combinatorial therapy could affect the pancreatic cancer cell line 6606PDA. These concentrations correspond to approximately 50% of the IC50 value of each compound for inhibiting the proliferation of 6606PDA cells. The addition of BKM120 significantly reduced the viability of these cells when grown on a flat surface (Fig. 1A and Additional file: Figure S1) or in a three-dimensional cell culture system (Fig. 1B). Furthermore, the addition of BKM120 enhanced the cytotoxic effects of BI-3406 and trametinib combination therapy, although this difference was only significant in the three-dimensional cell culture system (Additional file: Figure S2). At 1µM, the concentration used for all in vitro experiments, BKM120 inhibited PI3K signalling, as indicated by reduced phosphorylation of p70 S6 kinase (Additional file: Figure S3).

**Fig. 2:**
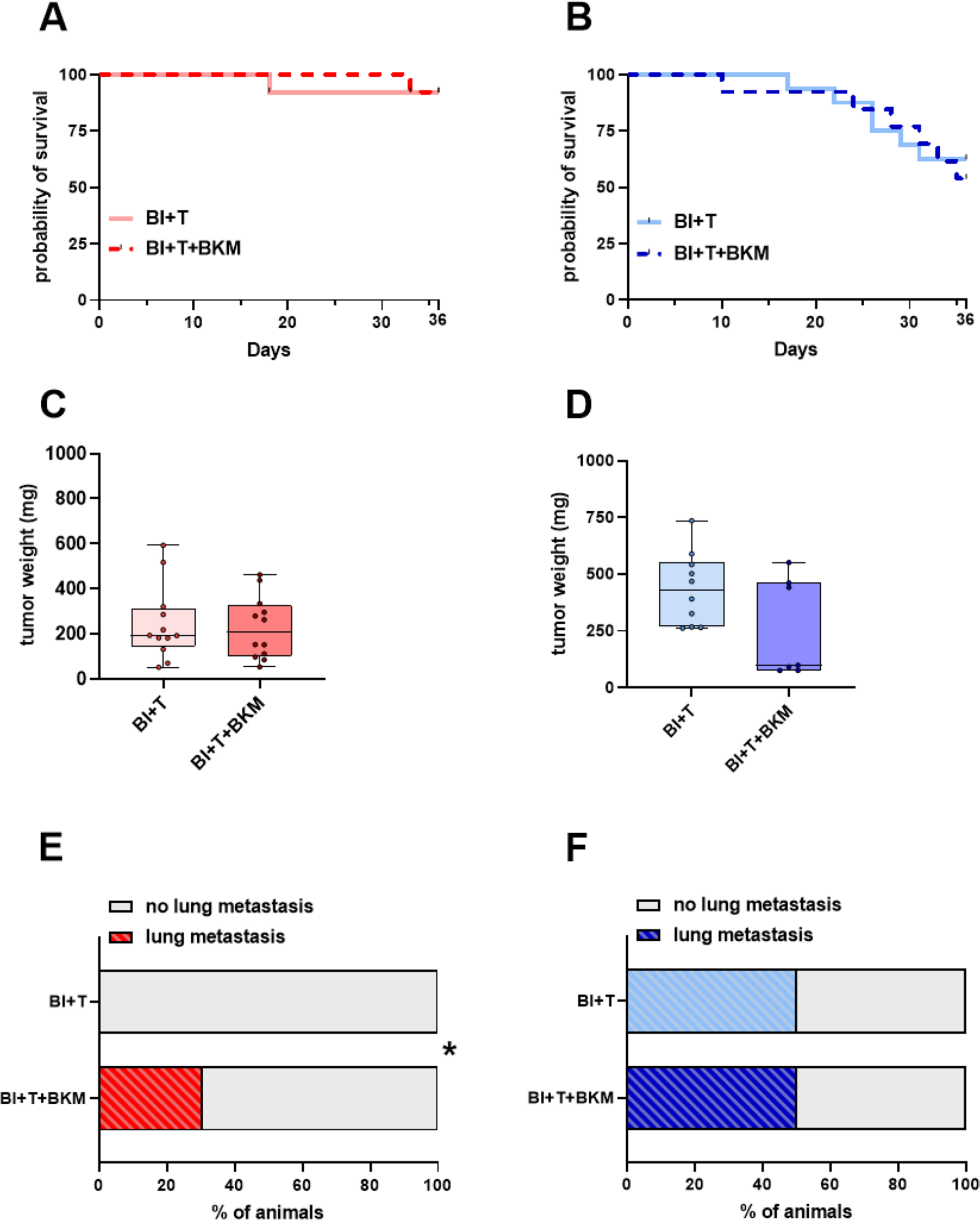
Influence of combinatorial therapies on survival, tumor weight, and metastasis. Female (**A**, **C**, **E**) and male (**B**, **D**, **F**) mice in a syngeneic pancreatic cancer model were assessed for survival (**A**, **B**), tumor weight (**C**, **D**), and the presence of histologically detectable lung metastases (**E**, **F**) following treatment with the BI-3406 (BI) and trametinib (T) combination therapy with or without BKM120 (BKM). Statistical analyses were performed using the Kaplan–Meier estimator with log-rank test (**A**, **B**), unpaired t-test (**C**), Mann–Whitney test (**D**), or Fisher’s exact test (**E**, **F**). *p < 0.05

In a syngeneic pancreatic cancer animal model, the efficacy of BKM120 combined with BI-3406 and trametinib was compared to the dual therapy (BI-3406 and trametinib). Both treatment regimens demonstrated similar survival outcomes in male and female mice (Fig. 2A and B). Tumor weights were comparable in female mice following both therapies (Fig. 2C). In male mice only a non-significant reduction was observed in the triple therapy group compared to the dual therapy group (Fig. 2D).

Interestingly, no lung metastases were detected in female mice following dual therapy, while more than 40% of male mice developed lung metastases (Fig. 2E, F, and Additional file: Figure S4). The addition of BKM120 to BI-3406 and trametinib significantly increased lung metastases in female mice, whereas no significant change was observed in male mice (Fig. 2E and F).

**Fig. 3:**
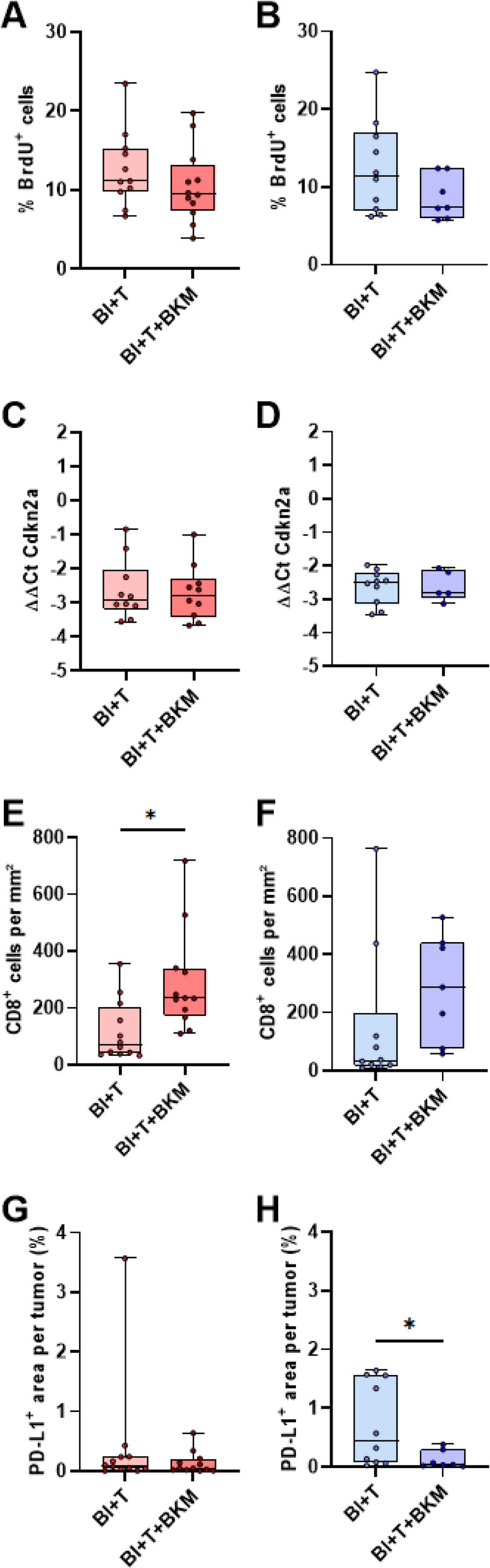
Effect of combinatorial therapies on tumor biology. Primary tumors from female (**A**, **C**, **E**, **G**) and male (**B**, **D**, **F**, **H**) mice were analyzed for BrdU⁺ proliferating cells (**A**, **B**), Cdkn2a expression (**C**, **D**), CD8⁺ T-cell infiltration (**E**, **F**), and PD-L1⁺ tumor area (**G**, **H**). Mice were treated with the BI-3406 (BI) and trametinib (T) combination therapy with or without BKM120 (BKM). Statistical analyses were performed using an unpaired t-test (**A**–**D**) or Mann–Whitney test (**E**–**H**). *p < 0.05

To characterize the tumors, we analyzed cancer cell proliferation (Additional file: Figure S5), *Cdkn2a* expression as a marker of senescence, the number of CD8^+^ cells (Additional file: Figure S6), and programmed cell death ligand 1 (PD-L1 officially named CD274) abundance (Additional file: Figure S7), in the tumor. The percentage of BrdU-positive cells was non-significantly reduced following BKM120 + BI-3406 + trametinib therapy compared to BI-3406 + trametinib alone in both female and male mice (Fig. 3A, B). No significant differences in *Cdkn2a* expression were observed between these therapies in either sex (Fig. 3C, D). However, a higher number of CD8^+^ cells was detected in the BKM120 + BI-3406 + trametinib group compared to the BI-3406 + trametinib group (Fig. 3E, F), although this difference was non-significant in male mice. When assessing PD-L1 expression in tumors, female mice exhibited only non-significant changes in protein abundance, whereas male mice showed a significant reduction (Fig. 3G, H).

**Fig. 4:**
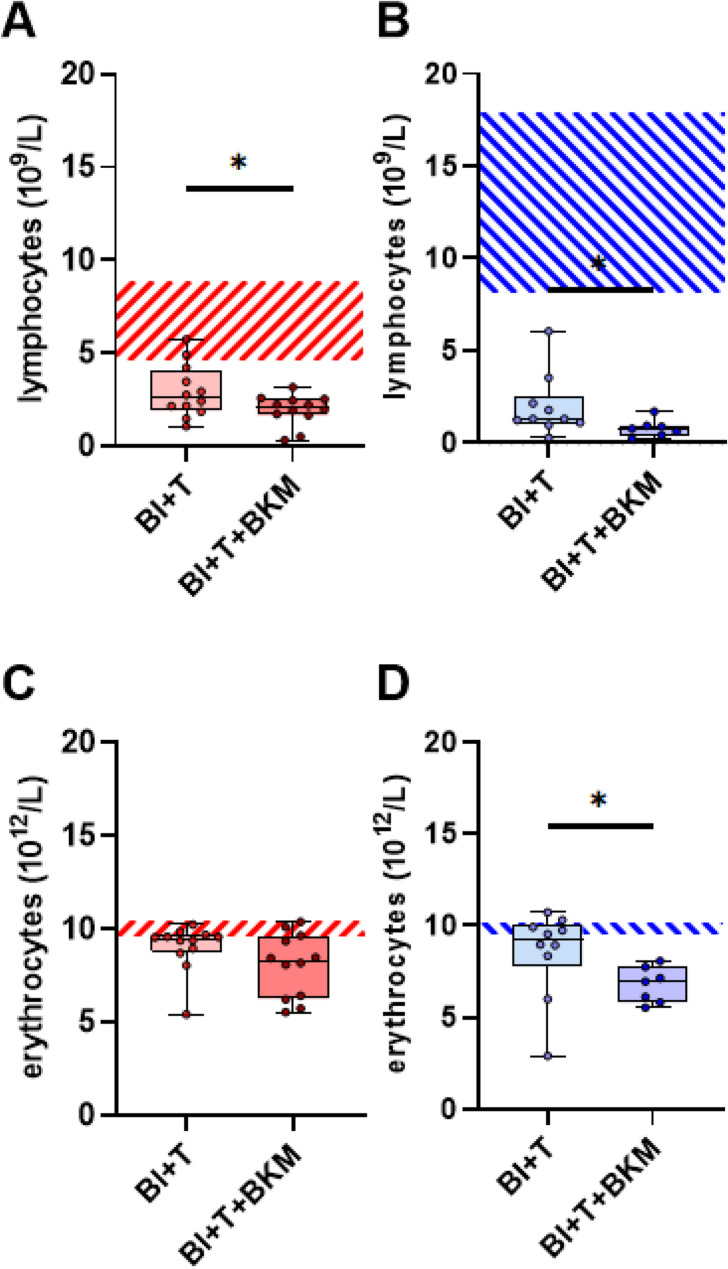
Impact of combinatorial therapies on blood cell counts. Blood from female (**A**, **C**) and male (**B**, **D**) mice was analyzed for lymphocytes (**A**, **B**) and erythrocytes (**C**, **D**) following treatment with the BI-3406 (BI) and trametinib (T) combination therapy with or without BKM120 (BKM). Dashed areas indicate the range observed in healthy C57BL/6 mice (n = 5). Statistical analyses were performed using an unpaired t-test (**A**) or Mann–Whitney test (**B**–**D**). *p < 0.05

To evaluate potential adverse effects on the general health of the animals, blood cell counts were measured, animal distress was assessed, and levels of AST, creatinine, and C-peptide were analyzed in blood plasma to detect potential liver or kidney damage or endocrine pancreas dysregulation. The addition of BKM120 to the BI-3406 and trametinib combination therapy significantly reduced lymphocyte counts in both female and male mice (Fig. 4A, B). It also reduced erythrocyte counts in female and male mice (Fig. 4C, D). This decrease was significant in male, but not significant in female mice.

**Fig. 5:**
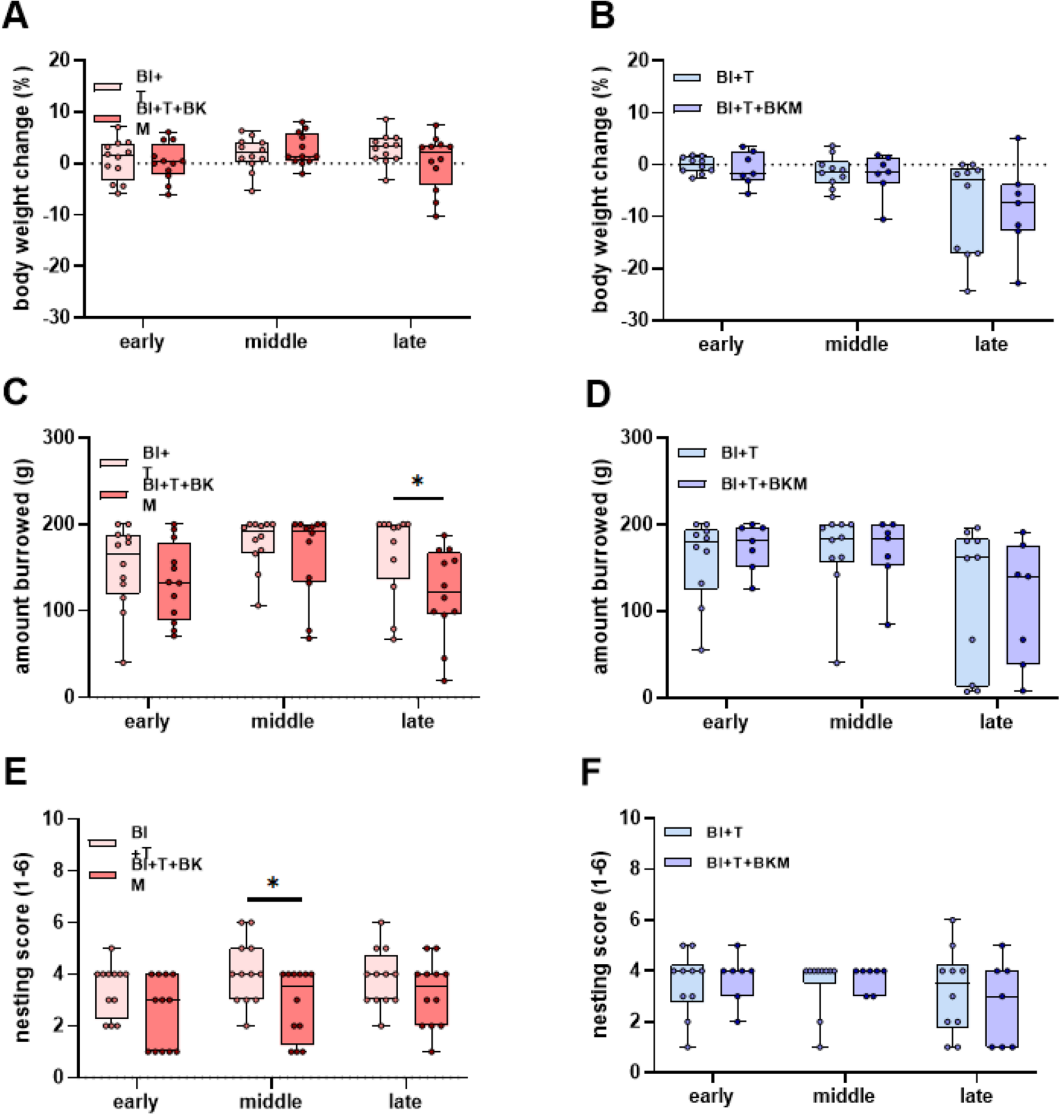
Impact of combinatorial therapies on mouse wellbeing. Female (**A**, **C**, **E**) and male (**B**, **D**, **F**) mice were assessed for body weight changes (**A**, **B**), burrowing activity (**C**, **D**), and nesting behavior (**E**, **F**) during the early, middle, and late phases of the experiment following treatment with the BI-3406 (BI) and trametinib (T) combination therapy with or without BKM120 (BKM). Statistical analyses were performed using two-way repeated measures ANOVA with Sidak’s post-hoc test (**A**–**F**). *p < 0.05

**Fig. 6:**
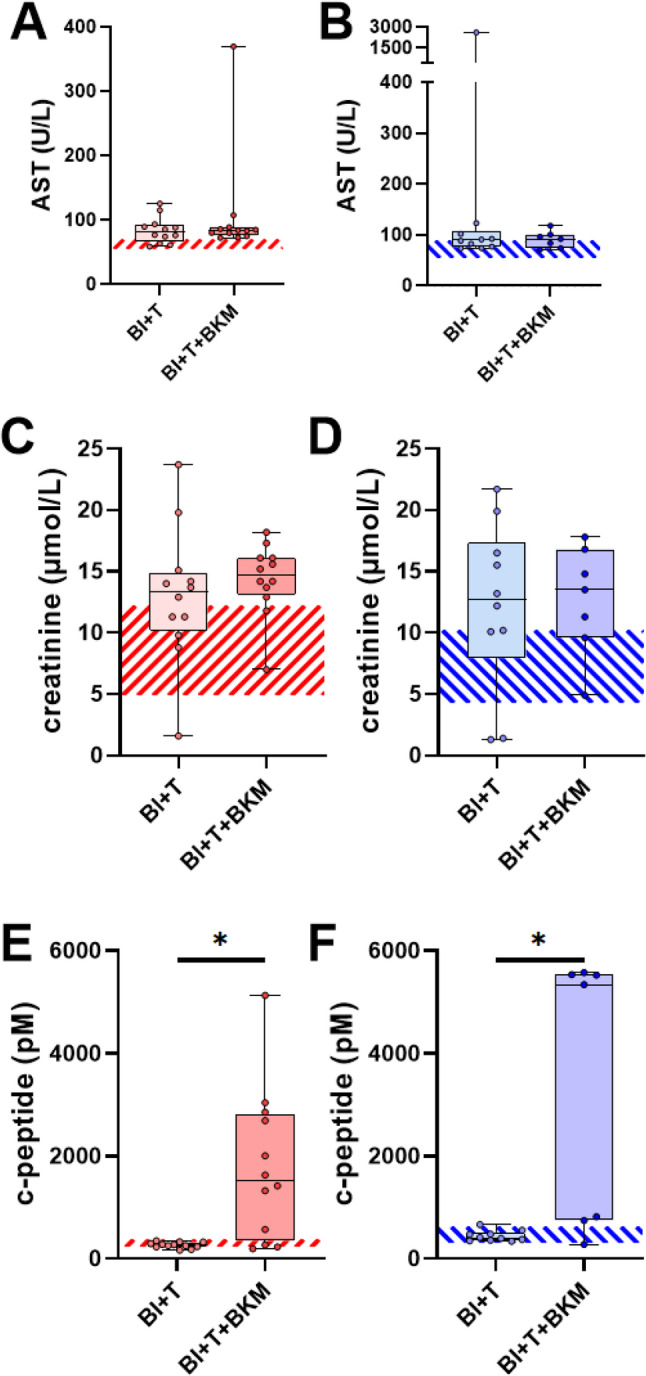
Effect of combinatorial therapies on blood plasma parameters. Blood plasma from female (**A**, **C**, **E**) and male (**B**, **D**, **F**) mice was analyzed for AST activity (**A**, **B**), creatinine concentrations (**C**, **D**), and C-peptide levels (**E**, **F**) following treatment with the BI-3406 (BI) and trametinib (T) combination therapy with or without BKM120 (BKM). Shaded areas indicate the range observed in healthy C57BL/6 mice (n = 5). Statistical analyses were performed using an unpaired t-test (**C**–**E**) or Mann–Whitney test (**A**, **B**, **F**). *p < 0.05

When assessing whether BKM120 caused additional distress to the mice, only a few significant differences between treatment groups were observed in body weight, burrowing activity, or nesting behavior (Fig. 5A–F). For example, adding BKM120 significantly reduced burrowing activity in female mice during the late phase and nesting behavior during the middle phase of the experiment (Fig. 5C and E). While BKM120 had minimal, non-significant effects on AST and creatinine concentration (Fig. 6A-D), it significantly increased C-peptide levels in female and male mice (Fig. 6E and F).

## Discussion

The results of this study provide limited evidence supporting the hypothesis that adding BKM120 to the BI-3406 and trametinib combination therapy enhances anti-cancer effects. While in vitro experiments demonstrated that adding BKM120 significantly reduced pancreatic cancer cell viability and increased cytotoxicity (Fig. 1, Additional file: Figure S2), the in vivo impact on tumor size was minimal (Fig. 2C-D).

The hypothesis was raised whether pancreatic cell lines that are less sensitive to RAS inhibition might show a stronger response to the triple combination. While we did not address this question in the present project, our collaboration partners have previously published evidence inconsistent with this hypothesis. Specifically, adding BKM120 to trametinib and BI-3406 chemotherapy led to a non-significant, small reduction in biomass and proliferation in CAPAN-1 cells, which have low sensitivity to RAS inhibition. In contrast, a significant reduction in biomass was observed in MIA PaCa-2 cells, which are more sensitive to RAS inhibition (Figs. 1, 2 and 3) [17]. These data suggest that PDAC cell lines with low sensitivity to RAS inhibition do not necessarily exhibit a stronger response to the triple combination therapy.

At the same time, several findings highlighted significant drawbacks of adding BKM120 to the BI-3406 and trametinib combination therapy (Figs. 2E, 4, 5C and E and 6E-F). Some of these adverse effects have previously been observed with BKM120 as monotherapy or in combination with other drugs. For instance, lymphopenia was observed as an adverse effect in preclinical studies of glioblastoma treatment with BKM120 [21]. Similarly, hyperinsulinemia was reported in the same study [21]. However, several methodological limitations must be considered regarding our toxicity assessment. Our evaluation was primarily based on behavioural observations, limited hematology, and selected biochemical parameters, whereas standard preclinical toxicology protocols typically incorporate more extensive hematology, broader clinical chemistry, and systematic histopathological examinations of major organs [22]. In addition, we employed a fixed dose and dosing schedule, although preclinical and clinical evidence indicates that both dose and schedule can substantially influence toxicity outcomes [22, 23]. Accordingly, variations in dosage or dosing regimen might mitigate the adverse effects observed in our study.

Behavioural indicators, such as reduced burrowing and nesting activity, potentially reflecting fatigue, have also been associated with BKM120 therapy in cancer patients [24, 25]. In our experiments, the combination of BKM120, BI-3406, and trametinib also led to a significant increase in lung metastases in female mice, whereas this effect was not observed in male mice (Fig. 2E, F). Such increased lung metastases is often induced as a paradoxical reaction to various chemotherapies [26]. However, combined inhibition of PI3K and MEK by ST-162 reduced metastases in a melanoma mouse model, while PI3K inhibition with wortmannin also suppressed metastasis in esophageal squamous cell carcinoma, rather than inducing it [27, 28]. Thus, the observed induction of metastasis might be specific to the combinatorial use of BKM120, BI-3406, and trametinib rather than a typical effect of PI3K inhibition. Compared with females, males already exhibited increased lung metastases when treated with vehicle or dual therapy (Fig. 1E–F) [18], suggesting that male hormones may promote metastatic progression. Consistently, androgens have been reported to disrupt cell adhesion and enhance tumor cell migration and invasion in melanoma [29]. Furthermore, androgens have been shown to facilitate liver metastasis formation [30].

We also observed significantly higher CD8⁺ T-cell infiltration following triple therapy in females (Fig. 3E). This finding is consistent with previous reports of stronger CD8⁺ responses in females [31–35]. Moreover, androgen signaling has been implicated in the suppression of CD8⁺ T-cell function [33–35]. However, sex-specific differences in chemotherapy-induced CD8⁺ infiltration remain poorly characterized. Whether this observation can be reproduced in other studies and holds therapeutic relevance remains to be determined.

In our study, male tumors exhibited reduced PD-L1 expression following triple therapy (Fig. 3H). Given that PD-L1 expression on immune cells has been associated with favorable outcomes in various cancers [36–39], lower PD-L1 levels in male mice could potentially enhance the efficacy of triple therapy in this group. Nonetheless, these findings require validation in additional animal models before any therapeutic relevance can be inferred.

Orthotopic implantation and tail vein injection of pancreatic cancer cells were performed in the same mice. Although uncommon, this dual-model design enabled simultaneous assessment of chemotherapy effects on primary and metastatic tumors, reducing animal use and optimizing the balance between ethical considerations and scientific achievements. Moreover, since more than half of pancreatic cancer patients present with metastases at diagnosis [40], this approach reflects a clinically relevant scenario. We acknowledge, however, that potential interactions between primary and metastatic sites represent a limitation that should be considered and addressed in future studies. Another limitation of this study is that we were unable to directly demonstrate the efficacy of BKM120 within the tumors. To address this, we assessed p70 S6 kinase phosphorylation in vivo using immunohistochemistry. Phospho-p70 S6 kinase staining was observed in immune cells frequently localized at the tumor margins, as well as diffusely within tumor cells (Additional file: Figure S8). Despite the qualitative nature of immunohistochemistry, we quantified the percentage of tissue area positive for Phospho-p70 S6 kinase. Following BKM120 treatment, we observed a non-significant reduction in the stained area in female mice, whereas no reduction was detected in male mice (Additional file: Figure S8). Given that the median intratumoral concentration of BKM120 in tumor tissue (males: 2.1 µM, 5–95% CI: 0.68–3.6 µM; females: 2.4 µM, 5–95% CI: 0.41–2.4 µM) was approximately twofold higher than the effective concentration used in our in vitro experiments (1 µM) [18], these findings suggest that the in vivo dosage of BKM120 was sufficiently high within the tumor. Therefore, the limited effect observed on Phospho-p70 S6 kinase staining may reflect the presence of resistance mechanisms after 32 days of chemotherapy.

The observed limited efficacy, along with well-documented adverse side effects, raises one critical question: How likely is it that a combined inhibition of RAS/SOS1, MEK, and PI3K could evolve into an effective cancer therapy?

A first assessment to address this question can be based on clinical and preclinical studies describing the effectiveness of inhibiting two of the three potential drug targets.

Previous research has largely focused on combining PI3K inhibitors with MEK inhibitors. For instance, promising in vitro evidence for the beneficial effects of adding BKM120 to MEK inhibitors has been reported [41–43]. Similar findings have been made in animal models of lung and pancreatic cancer [43, 44]. Although a small early clinical study suggested the anti-tumor potential of combined MEK and PI3K inhibition [45], subsequent trials demonstrated limited anti-tumor efficacy in patients with advanced solid tumors [46–48].

In contrast, few preclinical studies have investigated the combination of MEK inhibitors with SOS1 inhibitors. Co-targeting SOS1 and MEK has shown enhanced anti-tumor responses both in vitro and in various animal models [8, 49, 50]. Notably, a phase 1 clinical trial (NCT04111458), initiated in 2019, is currently evaluating SOS1 inhibitors both as monotherapy and in combination with trametinib for patients with advanced cancers. However, the trial is not yet complete, and the clinical implications of this combinatorial therapy thus remain undefined.

Some studies also suggest a synergistic effect of PI3K inhibitors with RAS/SOS1 inhibition in vitro and in animal models [51–54]. For example, combining the RAS targeting antibody inRas37 with BEZ-235, a PI3K inhibitor, significantly inhibited tumor growth in a mouse model of pancreatic cancer [51].

These studies provide encouraging initial results by demonstrating the effectiveness of inhibiting two of the three potential drug targets: RAS/SOS1, MEK, and PI3K. Thus, these findings suggest that a triple inhibition strategy targeting RAS/SOS1, MEK, and PI3K could hold significant promise as a potent cancer therapy.

This conclusion raises a second question: What strategies could be employed to enhance the therapeutic success of this combined inhibition? One approach could involve replacing the pan-PI3K inhibitor BKM120 with other pan-PI3K inhibitors or inhibitors targeting specific PI3K subtypes. This could potentially increase the efficacy of cancer treatment and reduce adverse side effects associated with BKM120. Over the past few years, five PI3K inhibitors have received FDA approval [55]. However, three of these inhibitors (duvelisib, idelalisib, and umbralisib) have been withdrawn, leaving only two FDA-approved PI3K inhibitors: Alpelisib, an orally bioavailable PI3K-alpha inhibitor, and copanlisib, which inhibits all four classes of PI3K isoforms, with a notable focus on the α and δ isoforms [55, 56]. Alpelisib is currently used in combination with fulvestrant for the treatment of advanced metastatic breast cancer [57], while copanlisib is prescribed for the third-line treatment of follicular lymphomas but failed in combination with rituximab to benefit patients with relapsed indolent non-Hodgkin lymphoma [58, 59]. Moreover, adverse side effects associated with this new generation of PI3K inhibitors remain a challenge. For instance, alpelisib has been associated with significant adverse effects, including hyperglycemia, rash, and diarrhea [60, 61]. Copanlisib, on the other hand, has been reported to cause side effects such as hyperglycemia, hypertension, neutropenia, pneumonia, and diarrhea [62]. Given these limitations, there is still a clear need to evaluate novel inhibitors targeting PI3K.

Another strategy to improve efficacy and reduce adverse side effects of RAS, MEK, and PI3K inhibition could involve targeted drug delivery systems like nanoparticles. These systems may utilize the enhanced permeability and retention effect of tumors or employ active targeting mechanisms, such as selectively binding to EGFR-expressing cells [63].

## Conclusion

This study reveals that adding BKM120 to the combination of BI-3406 and trametinib provides negligible benefits while introducing considerable drawbacks. Therefore, this triple-drug regimen is not suitable for advancement to clinical trials. Considering trametinib’s well-established clinical efficacy and versatility in combination therapies, future efforts may achieve greater promise by exploring its use alongside next-generation agents such as alpelisib or BI 1,701,963 as potential anticancer strategies.

## Supplementary Information

Supplementary Material 1: Additional files Fig. S1-S8.

Supplementary Material 2.

## Data Availability

All data generated or analysed during this study are included in this published article [and its supplementary information files].

## Abbreviations

PDAC: Pancreatic ductal adenocarcinomas
PFA: Paraformaldehyde
