# Supplementary material for: Benefits and challenges of adding BKM120 to a BI-3406 plus trametinib combination therapy
Source: BMC Cancer. 2026 Jul 3;26:812. doi: 10.1186/s12885-026-16409-0 (PMC13332599; doi:10.1186/s12885-026-16409-0)
Supplement: Supplementary file 1 — Supplementary Material 1: Additional files Fig. S1-S8. [file 12885_2026_16409_MOESM1_ESM.zip › 12885_2026_16409_MOESM1_ESM/12885_2026_16409_MOESM1_ESM.pdf]

Figure S1

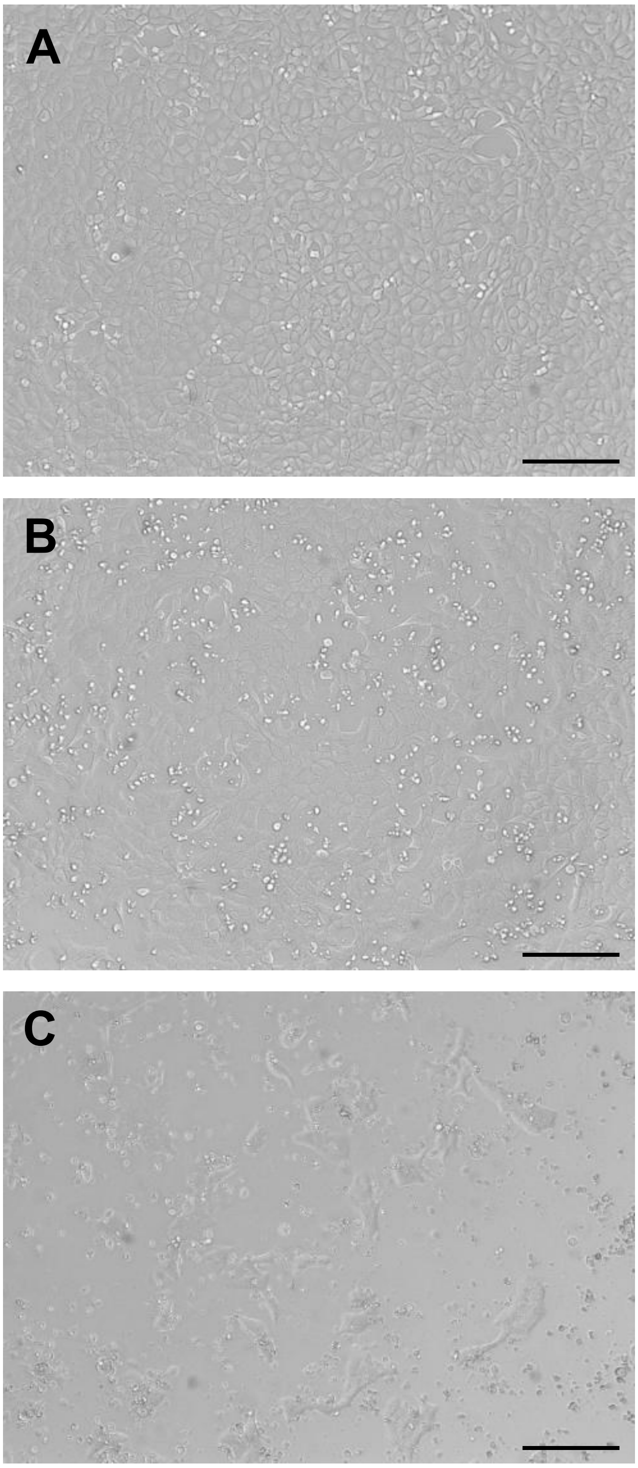

Figure S1. Impact of BKM120 on 6606PDA. Cells were treated with vehicle (A), 10 µM BI-3406 and 0.064 µM trametinib without (B) or with 1 µM BKM120 (C). Scale bar = 100 µm.
