# Supplementary material for: Benefits and challenges of adding BKM120 to a BI-3406 plus trametinib combination therapy
Source: BMC Cancer. 2026 Jul 3;26:812. doi: 10.1186/s12885-026-16409-0 (PMC13332599; doi:10.1186/s12885-026-16409-0)
Supplement: Supplementary file 1 — Supplementary Material 1: Additional files Fig. S1-S8. [file 12885_2026_16409_MOESM1_ESM.zip › 12885_2026_16409_MOESM1_ESM/12885_2026_16409_MOESM2_ESM.pdf]

**Figure S2**

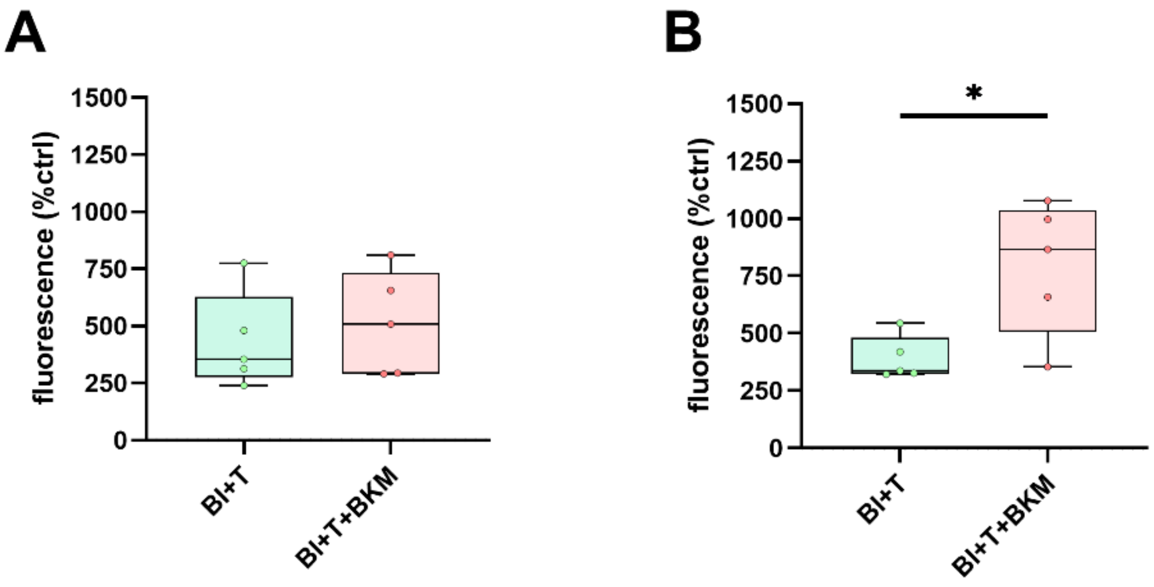


**Figure S2. Impact of BKM120 on cytotoxicity in combination therapy.** Cells were treated with BI-3406 (BI, 10 µM) and trametinib (T, 0.064 µM) with or without BKM120 (BKM, 1 µM) and analyzed for cytotoxicity in two-dimensional (A) and three-dimensional (B) culture systems. Statistical analyses were performed using an unpaired t-test. *$p < 0.05$
