# Supplementary material for: Benefits and challenges of adding BKM120 to a BI-3406 plus trametinib combination therapy
Source: BMC Cancer. 2026 Jul 3;26:812. doi: 10.1186/s12885-026-16409-0 (PMC13332599; doi:10.1186/s12885-026-16409-0)
Supplement: Supplementary file 1 — Supplementary Material 1: Additional files Fig. S1-S8. [file 12885_2026_16409_MOESM1_ESM.zip › 12885_2026_16409_MOESM1_ESM/12885_2026_16409_MOESM3_ESM.pdf]

Figure S3

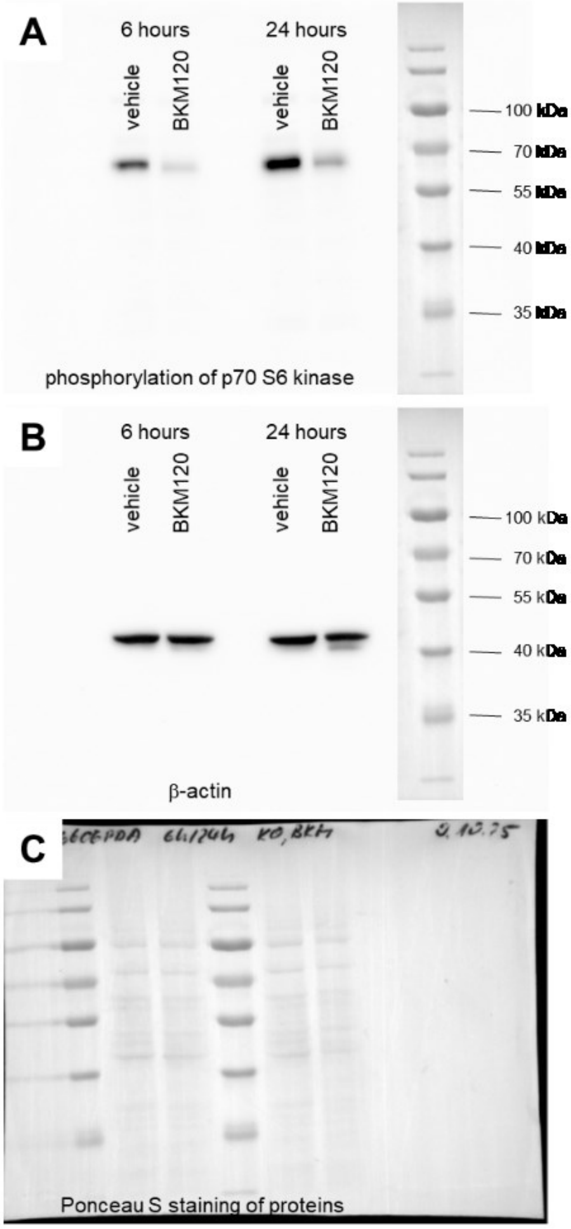

Figure S3. BKM120 inhibits phosphorylation of p70 S6 kinase. Cell extracts from 6606PDA cells treated with vehicle or 1 µM BKM120 for 6 h or 24 h were analyzed by Western blot using anti-p70 S6 kinase (phospho-T389) antibodies (A). Equal protein loading was verified by reprobing the same blot with anti-β-actin antibodies (B) and by Ponceau S staining of total protein (C).
