# Supplementary material for: Benefits and challenges of adding BKM120 to a BI-3406 plus trametinib combination therapy
Source: BMC Cancer. 2026 Jul 3;26:812. doi: 10.1186/s12885-026-16409-0 (PMC13332599; doi:10.1186/s12885-026-16409-0)
Supplement: Supplementary file 1 — Supplementary Material 1: Additional files Fig. S1-S8. [file 12885_2026_16409_MOESM1_ESM.zip › 12885_2026_16409_MOESM1_ESM/12885_2026_16409_MOESM4_ESM.pdf]

Figure S4

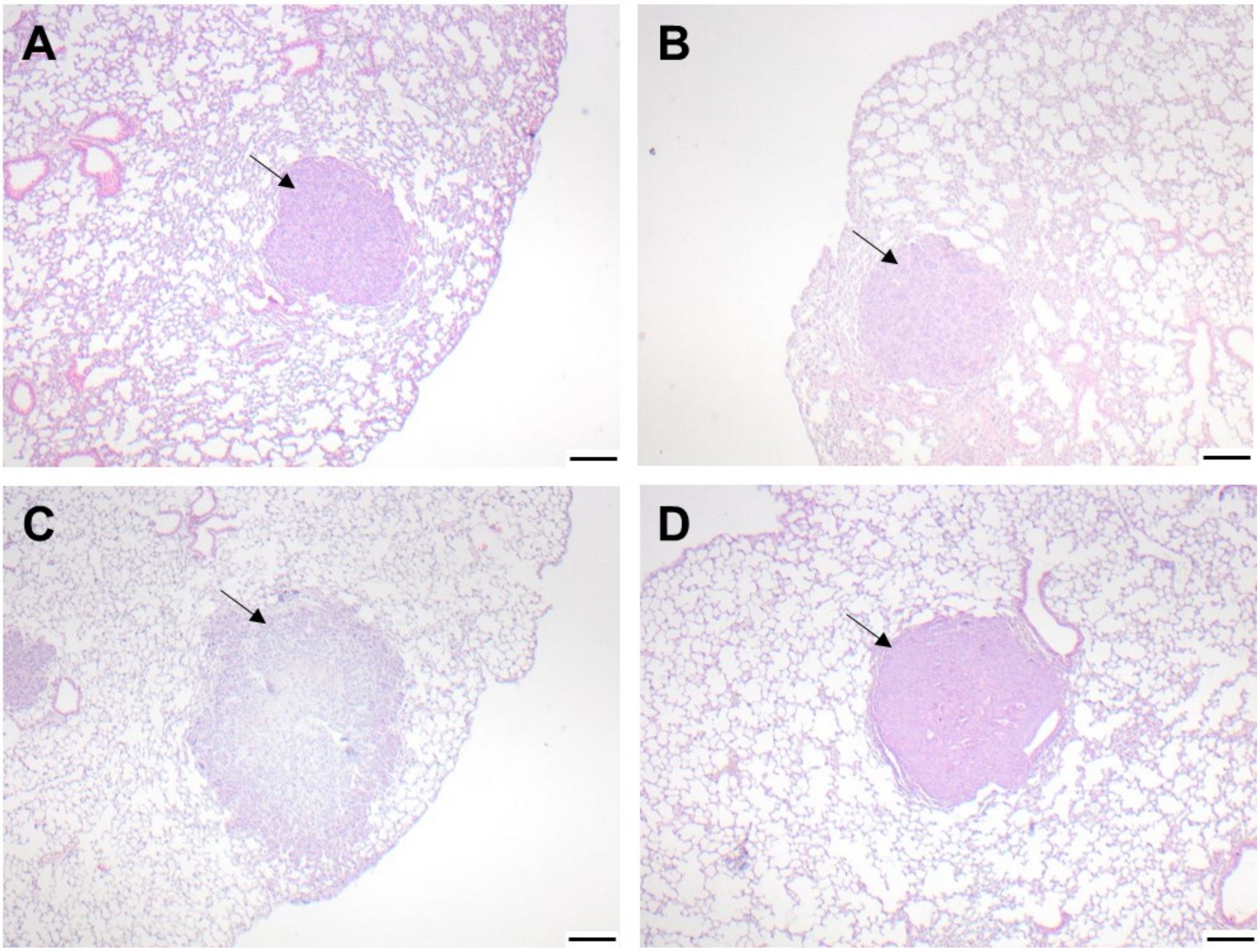

Figure S4. Hematoxylin and eosin–stained histological sections showing lung metastases. Representative examples of a lung metastasis (arrow) observed in a vehicle-treated male mouse (A), a male mouse treated with BI-3406 and trametinib (B), a male mouse treated with BI-3406, trametinib, and BKM120 (C), and a female mouse treated with BI-3406, trametinib, and BKM120 (D). Note that female mice treated with vehicle or BI-3406 plus trametinib did not develop metastases. Scale bar = 100 µm.
