# Supplementary material for: Benefits and challenges of adding BKM120 to a BI-3406 plus trametinib combination therapy
Source: BMC Cancer. 2026 Jul 3;26:812. doi: 10.1186/s12885-026-16409-0 (PMC13332599; doi:10.1186/s12885-026-16409-0)
Supplement: Supplementary file 1 — Supplementary Material 1: Additional files Fig. S1-S8. [file 12885_2026_16409_MOESM1_ESM.zip › 12885_2026_16409_MOESM1_ESM/12885_2026_16409_MOESM6_ESM.pdf]

**Figure S6**

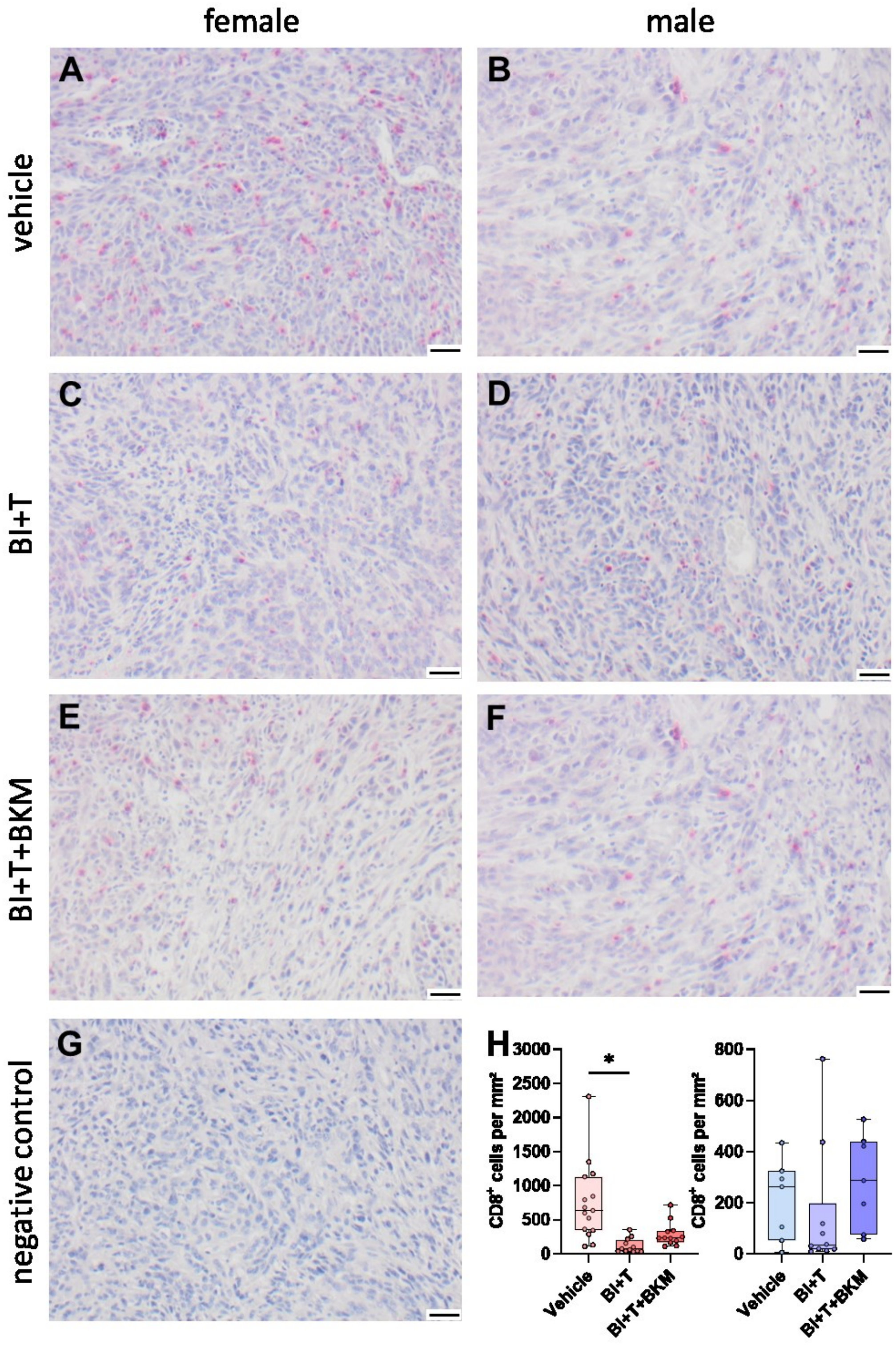


**Figure S6. CD8 immunohistochemistry.** CD8-positive cells (red) in pancreatic tumors following treatment with vehicle (A, B), BI-3406 plus trametinib (C, D), or BI-3406, trametinib, and BKM120 (E, F) in female (A, C, E) or male (B, D, F) mice and quantification (H) in female (red columns on the left) and male (blue columns on the right) mice. A negative control for immunohistochemistry (without primary antibody) is shown in (G). All tissue sections were counterstained with hematoxylin. Scale bar = 20 µm. Comparison to vehicle with Kruskal-Wallis & Dunn's post-hoc test: *$p < 0.05$.
