# Supplementary material for: Benefits and challenges of adding BKM120 to a BI-3406 plus trametinib combination therapy
Source: BMC Cancer. 2026 Jul 3;26:812. doi: 10.1186/s12885-026-16409-0 (PMC13332599; doi:10.1186/s12885-026-16409-0)
Supplement: Supplementary file 1 — Supplementary Material 1: Additional files Fig. S1-S8. [file 12885_2026_16409_MOESM1_ESM.zip › 12885_2026_16409_MOESM1_ESM/12885_2026_16409_MOESM8_ESM.pdf]

Figure S8

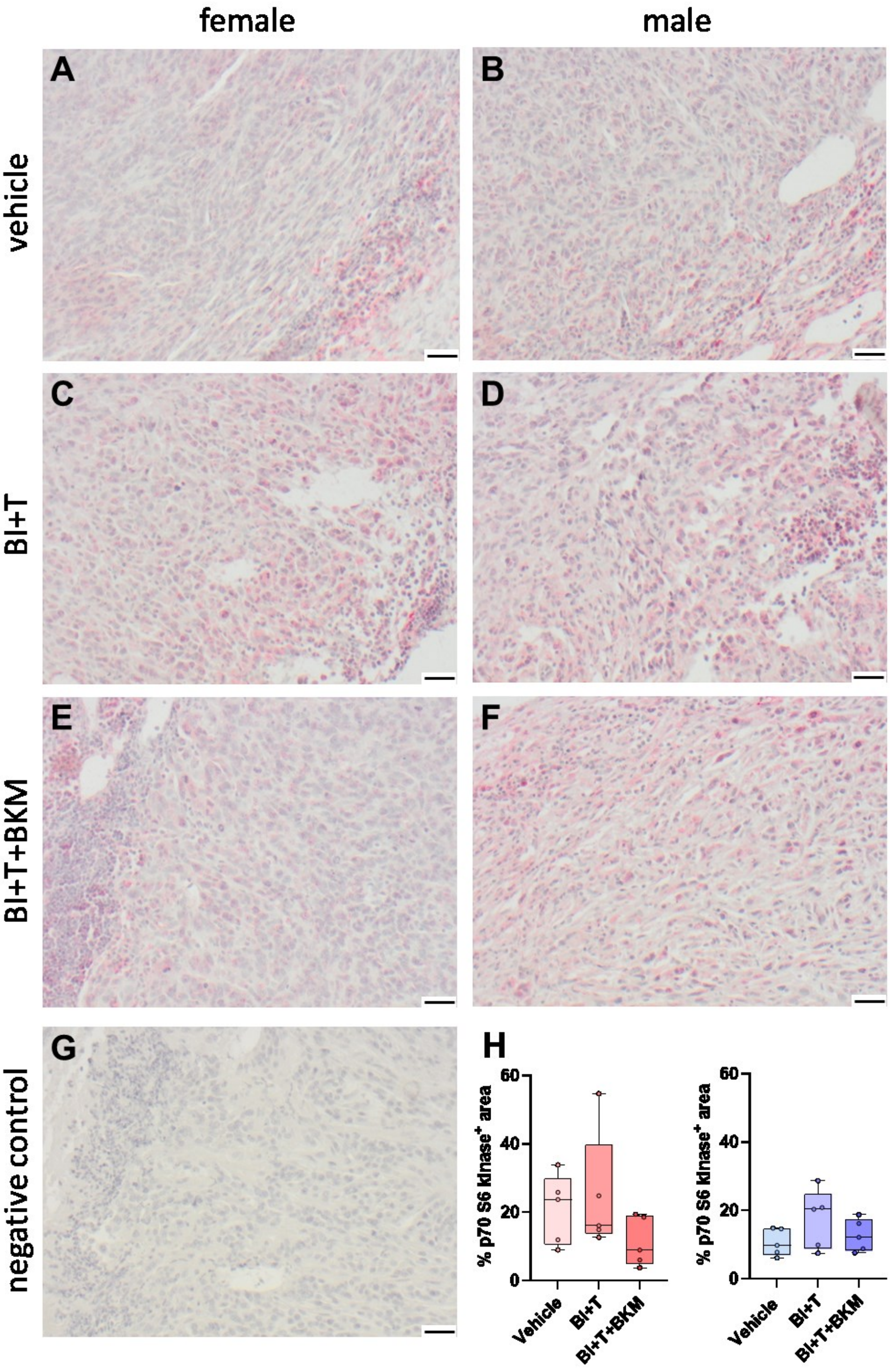

Figure S8. Immunohistochemistry staining of phosphorylated p70 S6 kinase. Cells positive for phosphorylated p70 S6 kinase (red) in pancreatic tumors after treatment with vehicle (A, B), BI-3406 plus trametinib (C, D), or BI-3406, trametinib, and BKM120 (E, F) in female (A, C, E) or male (B, D, F) mice and quantification (H) in female (red columns on the left) and male (blue columns on the right) mice. A negative control without primary antibody is shown (G). All tissue sections were weakly counterstained with hematoxylin. Scale bar = 20 µm. Comparison to vehicle with Kruskal-Wallis & Dunn's post-hoc test.
